# Supplementary material for: BoostMe accurately predicts DNA methylation values in whole-genome bisulfite sequencing of multiple human tissues
Source: BMC Genomics. 2018 May 23;19:390. doi: 10.1186/s12864-018-4766-y (PMC5966887; doi:10.1186/s12864-018-4766-y)
Supplement: Supplementary file 1 — Figure S1. Distribution of WGBS missingness across chromatin states, not normalized for total number of CpGs in that chromatin state. Figure S2. Distribution of distance from a WGBS or EPIC CpG to the nearest WGBS or EPIC CpG. Figure S3. CpG methylation pairwise differences as a function of distance genome-wide and in regions of higher across-tissue variance. Figure S4. CpG methylation pairwise differences for pancreatic islets as a function of distance genome-wide and in regions of higher across-tissue variance. Figure S5. Comparison of all CpGs and CpGs that had higher across-tissue variance. Figure S6. Joyplot showing distribution of WGBS methylation (beta) values within each chromatin state for all tissues. Figure S7. Performance at intermediate CpGs does not improve when using a balanced training distribution. Figure S8. Smooth scatterplot of beta values of CpGs shared between EPIC and WGBS. Figure S9. Imputation mitigates discordance between WGBS at EPIC at low WGBS depth regardless of EPIC probe type. Figure S10. Correlation among the top 30 features ranked by BoostMe. Figure S11. Correlation among the top 30 features ranked by random forests. Figure S12. Distribution of across-sample CpG variance values vs. number of missing values for each CpG. Table S1. Summary of the data used in this work. Table S2. Previously reported imputation metrics and those reported in this work. Table S3. All features included in BoostMe and random forests and their source. Table S4. Genome-wide performance of algorithms, trained on 500,000 CpGs, for predicting methylation values. Table S5. RMSE performance of BoostMe and random forests improves when training on continuous values. Table S6. Top 100 transcription factors ranked in descending order as reported by BoostMe, trained only using TFBS features. Table S7. Top 100 transcription factors ranked in descending order as reported by random forests trained only using TFBS features. (PDF 5703 kb) [file 12864_2018_4766_MOESM1_ESM.pdf]

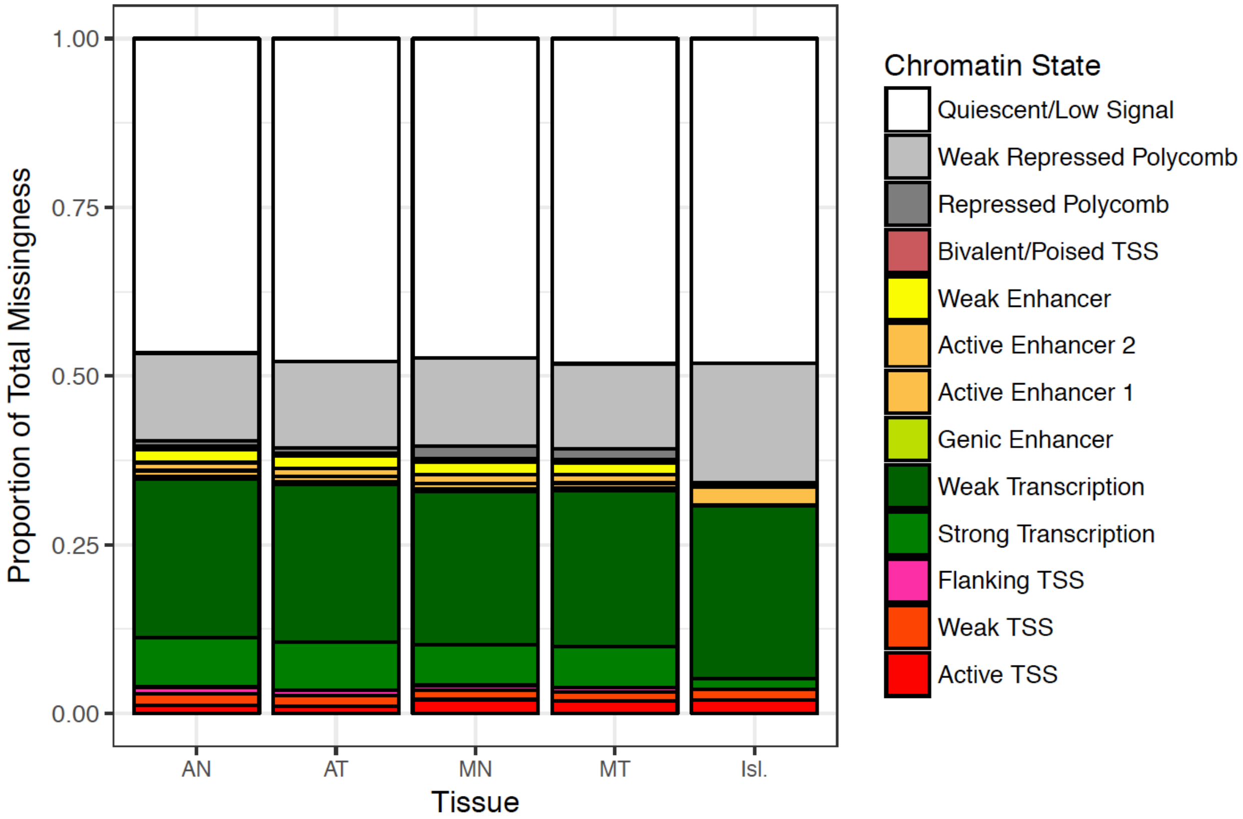

Figure S1: Distribution of WGBS missingness across chromatin states, not normalized for total number of CpGs in that chromatin state. The proportion of total missingness was calculated for each tissue as the number of missing CpGs (sequencing depth < 10x) in that chromatin state divided by the total number of missing CpGs in that tissue. AN = adipose NGT, AT = adipose T2D, MN = muscle NGT, MT = muscle T2D, Isl. = pancreatic islets. NGT = normal glucose tolerance, T2D = type 2 diabetic.

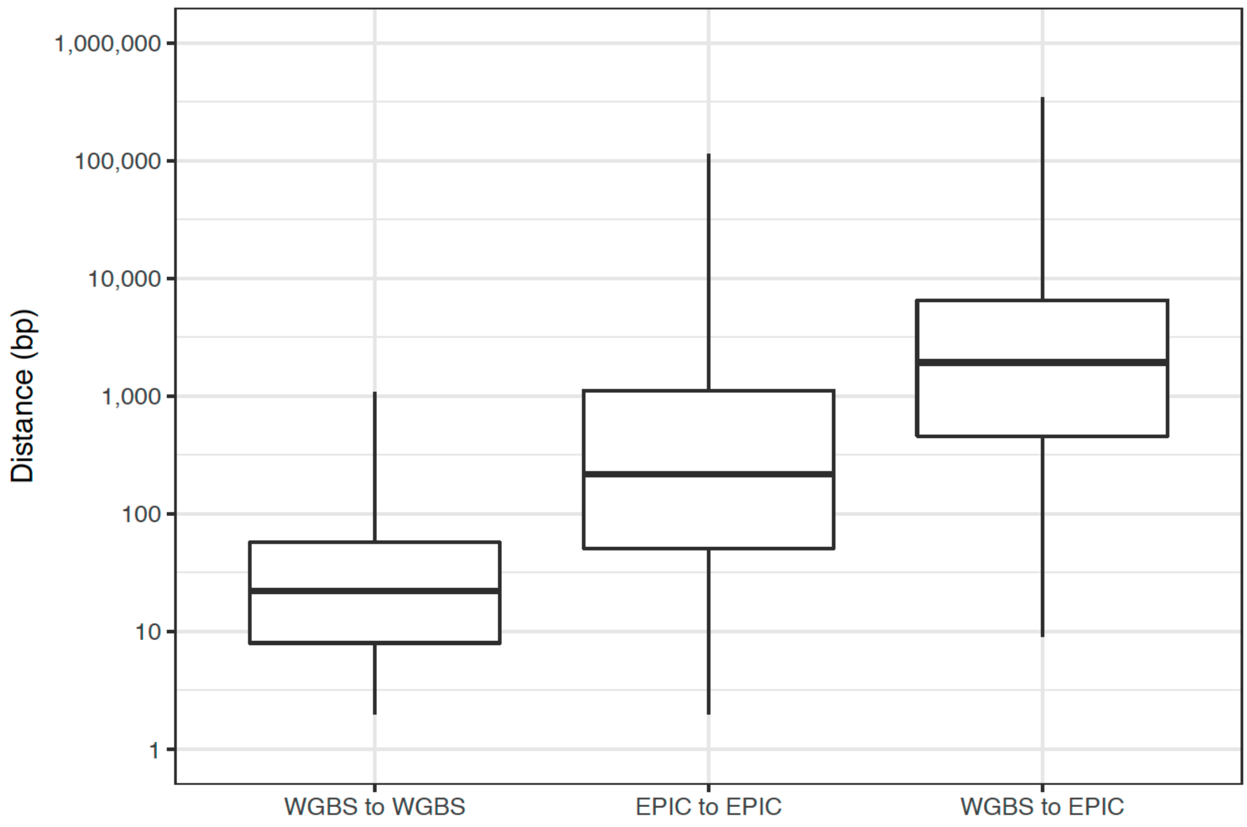

Figure S2: Distribution of distance from a WGBS or EPIC CpG to the nearest WGBS or EPIC CpG. WGBS to WGBS (W-W): distance from each WGBS CpG (n = ~25.5 million) to the nearest WGBS CpG. EPIC to EPIC (E-E): distance from each EPIC CpG (n = ~700,000 after quality control filtering) to the nearest EPIC CpG. WGBS to EPIC (W-E): distance from each WGBS CpG to the nearest EPIC CpG. All distances were calculated within autosomal chromosomes. Inner quartile ranges: W-W, 8-57 bp; E-E, 51-1,113 bp; W-E, 387-6,253 bp. Medians: W-W, 22 bp; E-E, 217; W-E, 1,799. Means: W-W, 50 bp; E-E, 1,534 bp; W-E, 8,240 bp.

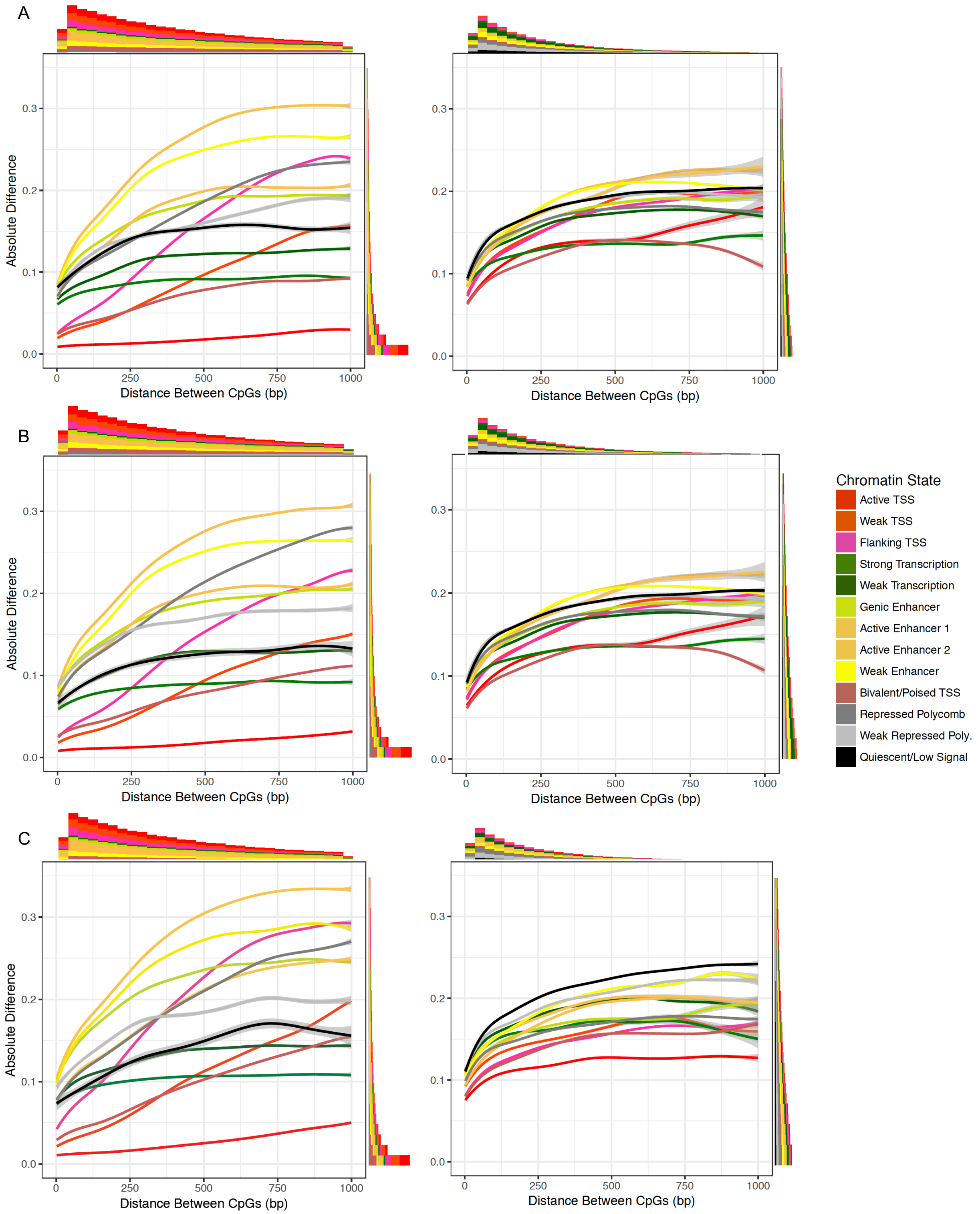

Figure S3: CpG methylation pairwise differences as a function of distance genome-wide (left) and in regions of higher across-tissue variance (right). (A) Adipose NGT, **(B)** Adipose T2D, **(C)** Muscle T2D.

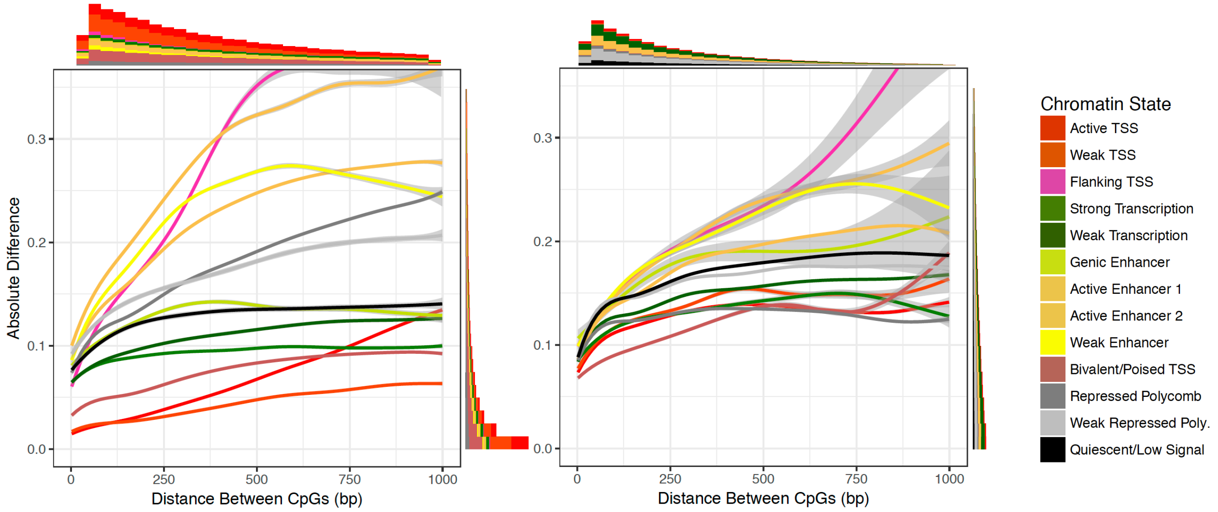

Figure S4: CpG methylation pairwise differences for pancreatic islets as a function of distance genome-wide (left) and in regions of higher across-tissue variance (right).

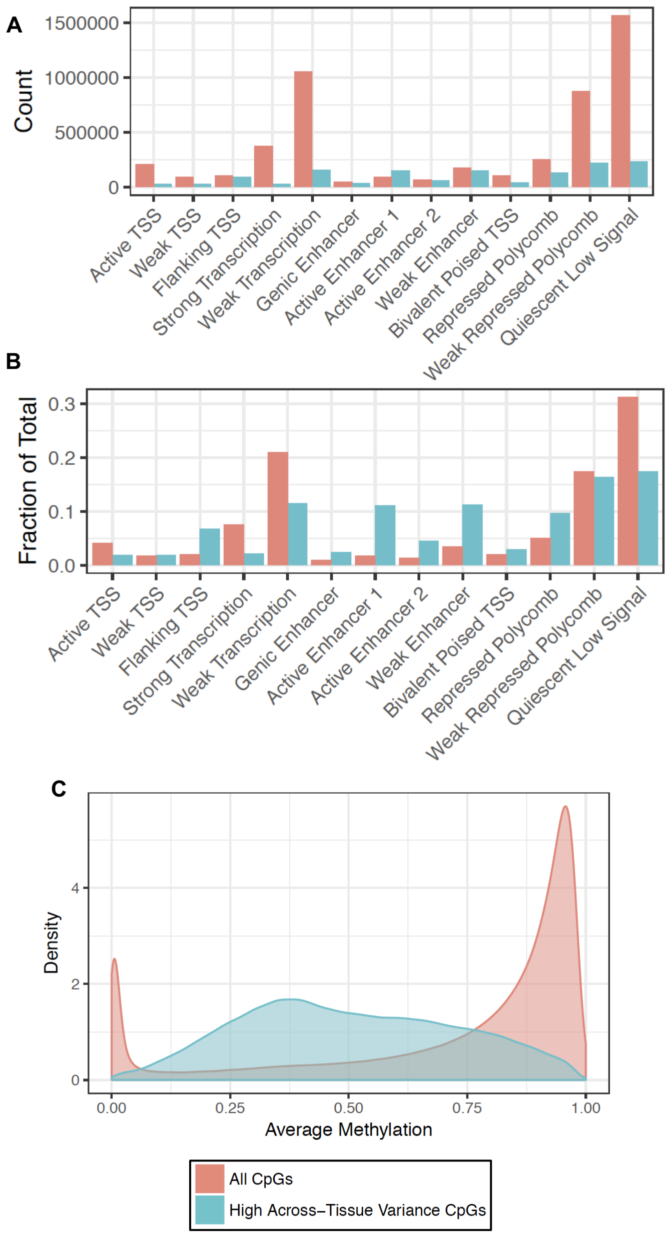

Figure S5: Comparison of all CpGs and CpGs that had higher across-tissue variance. (A) Raw counts of the number of CpGs in each chromatin state using a sample of 5 million CpGs (representative of all CpGs) and all ~1.3 million CpGs with higher across-tissue variance. CpGs were restricted to those that were able to be tested with BoostMe. **(B)** Same as data as (A) but normalized for total CpG count for each category (all CpGs vs. high across-tissue variance CpGs) to compare the relative enrichment of each chromatin state. **(C)** Methylation density distributions by average methylation across all samples within a tissue.

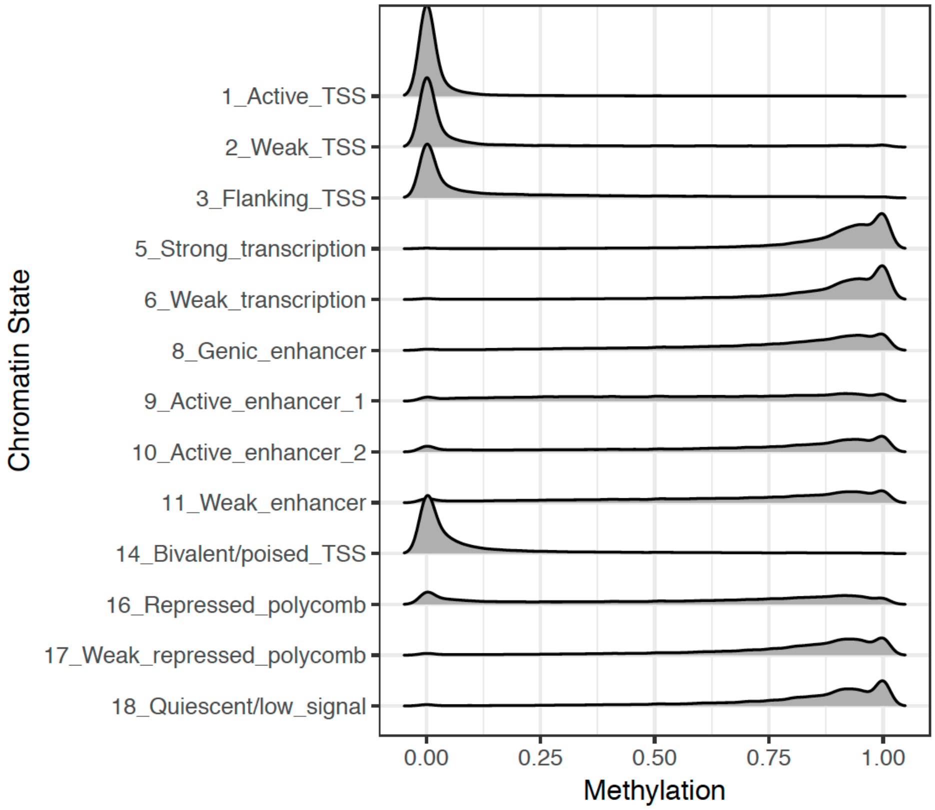

Figure S6: Joyplot showing distribution of WGBS methylation (beta) values within each chromatin state for all tissues.

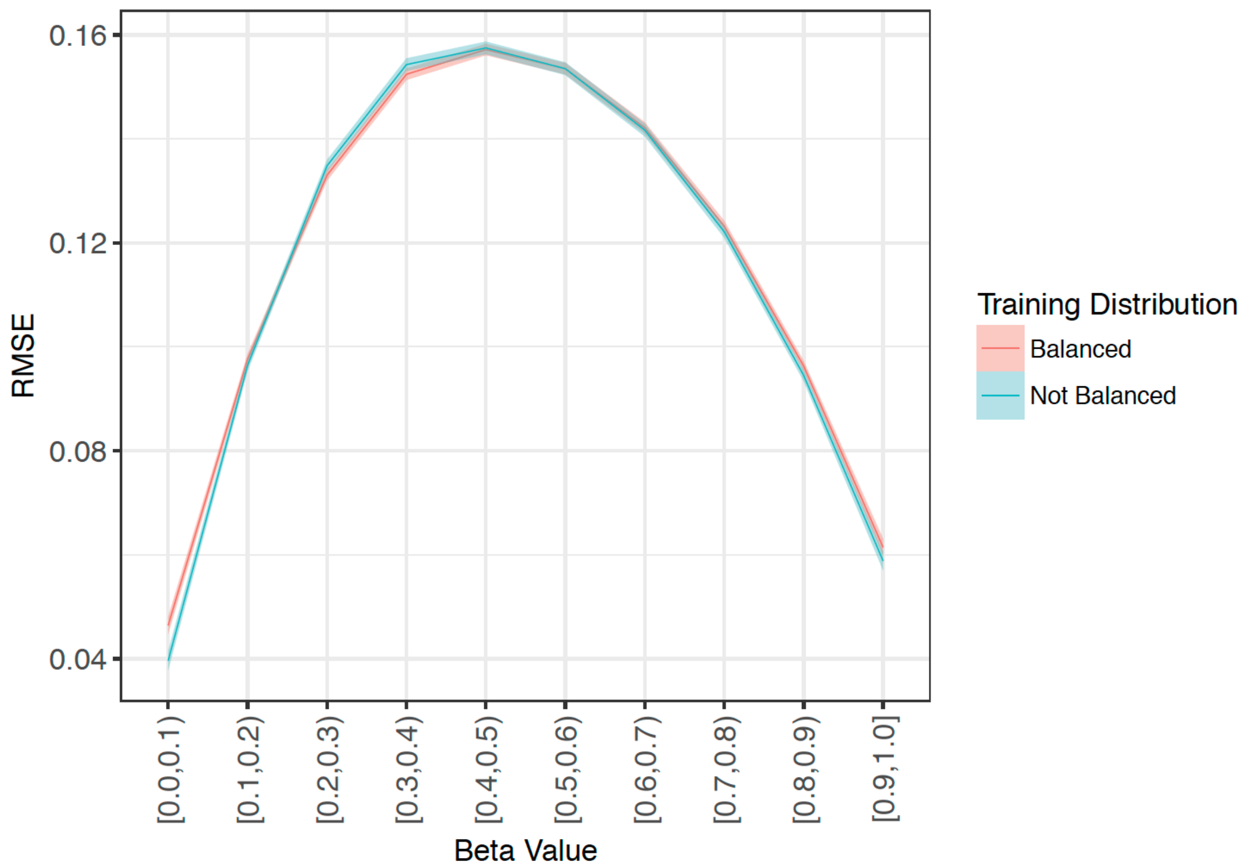

Figure S7: Performance at intermediate CpGs does not improve when using a balanced* training distribution. Root-mean squared error (RMSE) binned by the average WGBS beta value of a CpG (sample average) when training on a balanced (red) vs. not balanced (blue) training distribution. Shaded areas indicate 95% confidence interval (n=58). *The balanced training distribution was created by taking equal numbers of CpGs from each beta value bin (after binning into intervals of 0.1) for training.

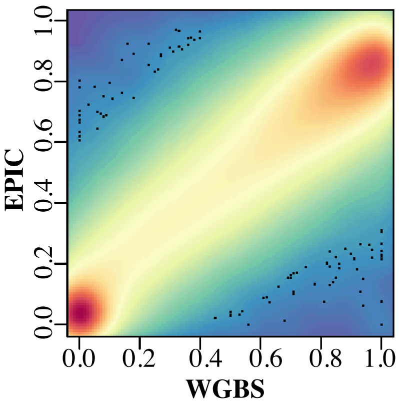

Figure S8: Smooth scatterplot of beta values of CpGs shared between EPIC and WGBS. x-axis, WGBS beta value; y-axis, EPIC beta value. $r^2 = 0.92$.

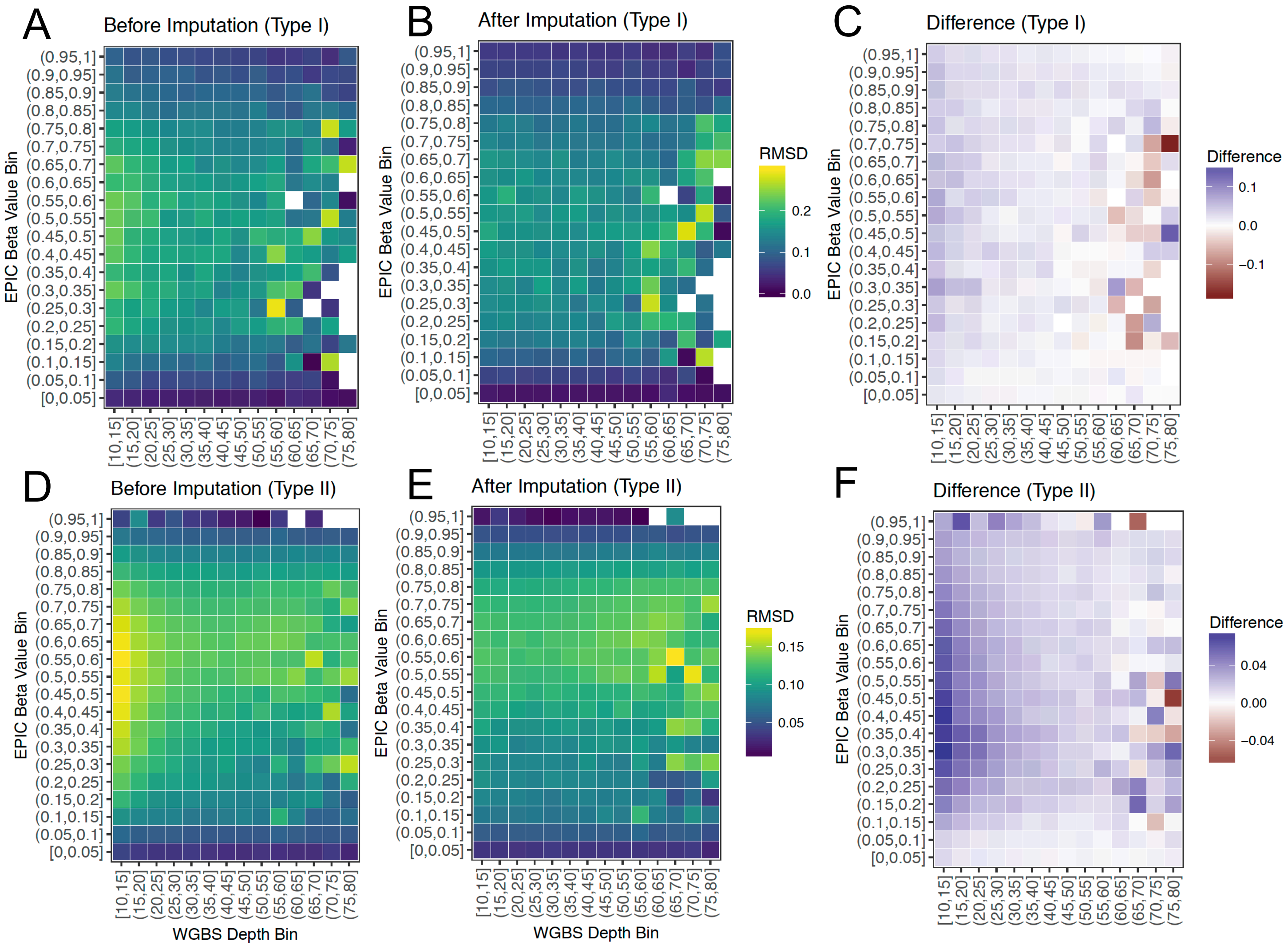

Figure S9: Imputation mitigates discordance between WGBS at EPIC at low WGBS depth regardless of EPIC probe type. (A) Root-mean squared discordance (RMSD) between EPIC and WGBS estimates at CpGs common between the two platforms, binned by WGBS depth of the CpG and EPIC beta value of the CpG for Type I EPIC probes only. **(B)** RMSD between EPIC and imputed WGBS values at the same CpGs as in A. **(C)** Difference between A and B. **(D-F)** Same as A-C for Type II EPIC probes only. Empty bins (white) indicate that no CpGs were present in that bin.

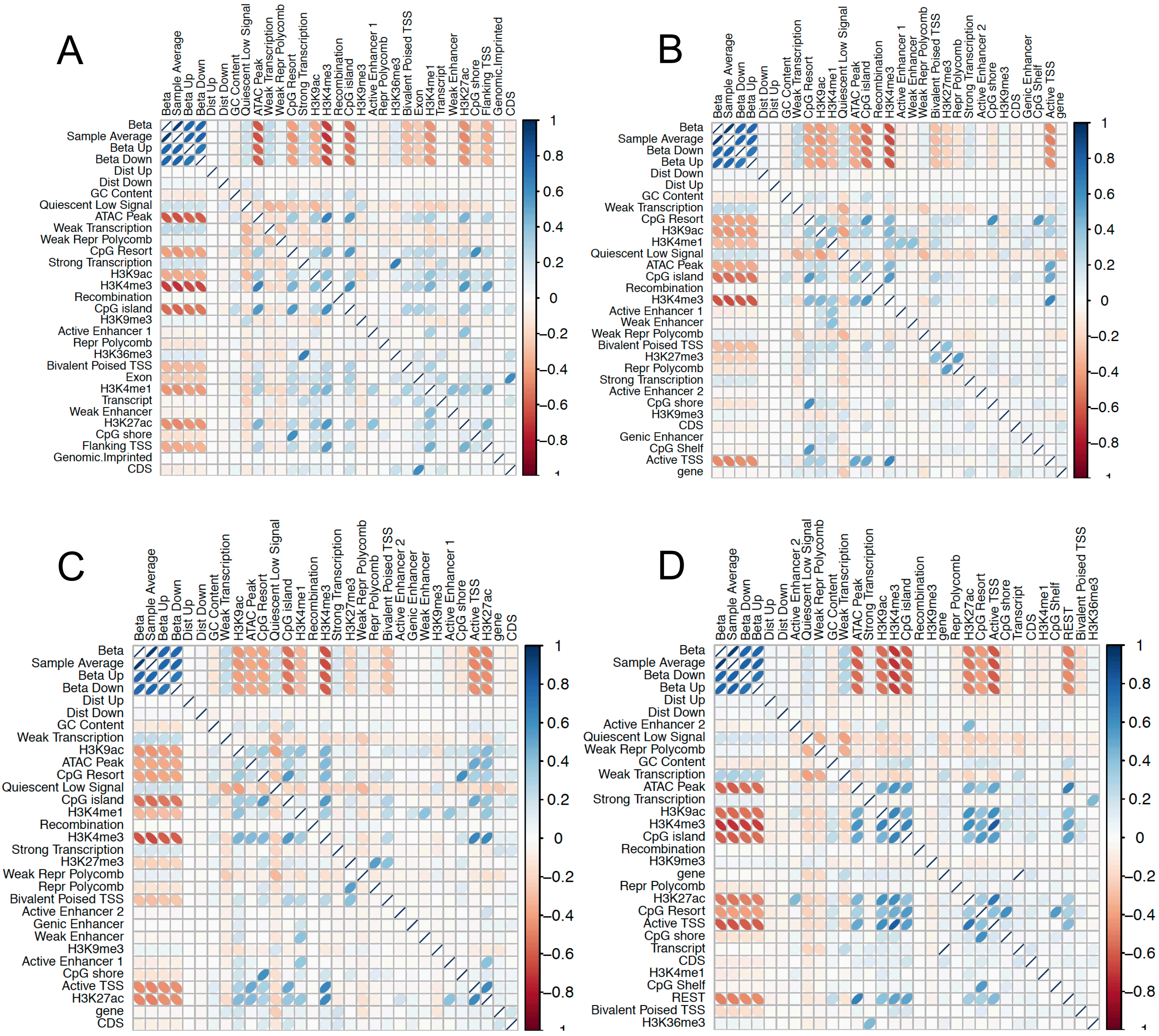

Figure S10: Correlation among the top 30 features ranked by BoostMe. (A) Adipose NGT, **(B)** Muscle NGT, **(C)** Muscle T2D, **(D)** Islet.

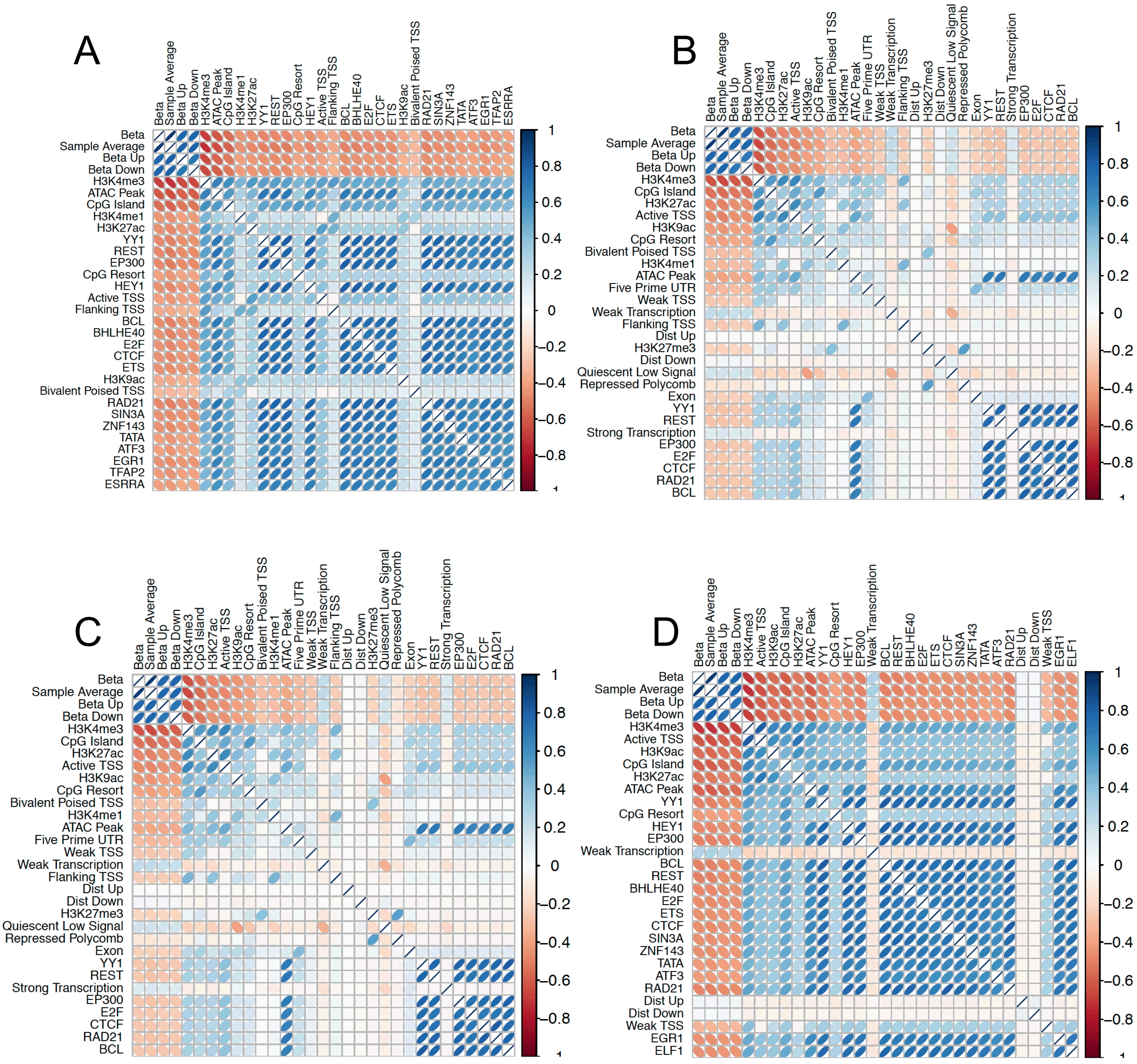

Figure S11: Correlation among the top 30 features ranked by random forests. (A) Adipose NGT, **(B)** Muscle NGT, **(C)** Muscle T2D, **(D)** Islet.

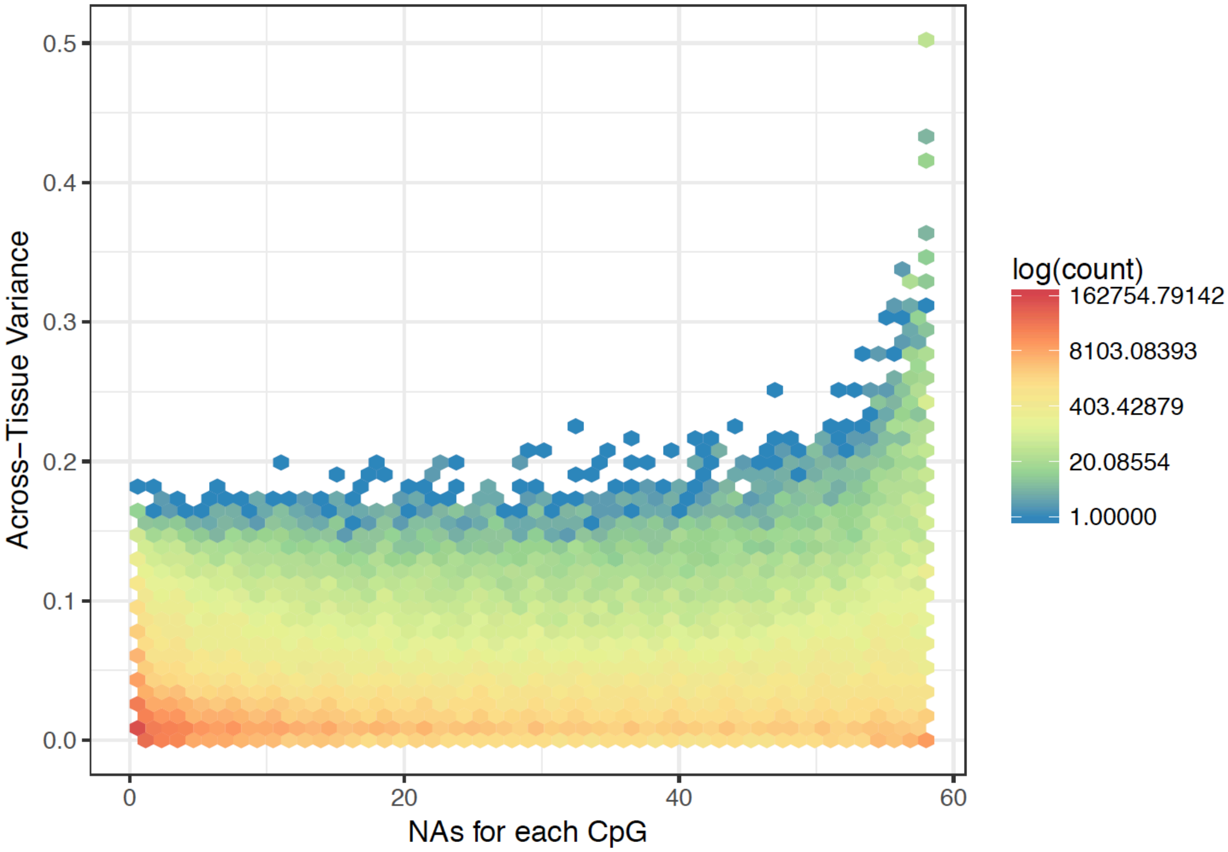

Figure S12: Distribution of across-sample CpG variance values vs. number of missing values (NAs) for each CpG. Given that variance is affected by sample size, a cutoff of 40 was chosen as the maximum number of missing values to consider the CpG a valid candidate for having high across-tissue variance.

Table S1: Summary of the data used in this work.

Tissue	T2D Status	Sample Size
Adipose	NGT	12
	T2D	12
Skeletal Muscle	NGT	12
	T2D	12
Pancreatic Islets	NGT	10

NGT = normal glucose tolerance, T2D = type 2 diabetic. Adipose NGT and muscle NGT were from the same 12 NGT patients; adipose T2D and muscle T2D were from the same 12 T2D patients.

Table S2: Previously reported imputation metrics and those reported in this work.

Method	Train Data/Test Data (if different); Tissue	RMSE	AUROC	AUPRC	Accuracy
RF [54]	Illumina-450k sites /WGBS; blood	0.23	-	-	0.92
Penalized Functional Regression [61]	Illumina-450k; blood	0.25	-	-	-
DNN (CpGenie) [59]	RRBS; GM12878 cell line	-	0.85	0.69	-
DNN (DeepCpG) [53]	scBS-seq; mouse ES cells	-	0.93	-	-
This work, DeepCpG	WGBS; adipose, muscle, islets	0.18	0.94	0.98	0.91
This work, RF, BoostMe	WGBS; adipose, muscle, islets	0.09	0.99	0.99	0.96

- = not reported. RF - random forests, DNN - deep neural network.

Table S3: All features included in BoostMe and random forests and their source.

Features	Source
Sample average<br>Upstream CpG beta<br>Downstream CpG beta	WGBS
GC content	UCSC Genome Browser
Recombination rate	HapMap (accessed from UCSC)
CpG island (CGI)<br>CpG shore<br>CpG shelf<br>CpG resort	GENCODE
ATAC peaks	ATAC-seq, macs2
H3K27ac<br>H3K27me3<br>H3K36me3<br>H3K4me1<br>H3K4me3<br>H3K9ac<br>H3K9me3	ChIP-seq, ChromHMM
AFP, AHR, AIRE, ALX1, ALX3, ALX4, AP1, AP3, ARHGEF12, ARID3A, ARID5A, ARID5B, ARNT, ARNTL, ARX, ASCL2, ATF2, ATF3, ATF4, ATF6, ATF7, ATOH1, BACH1, BACH2, BARHL1, BARHL2, BARX1, BARX2, BATF, BBX, BCL, BCL6B, BDP1, BHLHA15, BHLHE22, BHLHE23, BHLHE40, BHLHE41, BPTF, BRCA1, BSX, CACBP, CACD, CBX5, CCDC6, CCNT2, CDC5L, CDX, CDX1, CDX2, CEBPA, CEBPB, CEBPD, CEBPE, CEBPG, CENPB, CHD2, CLOCK, COMP1, CPEB1, CPHX, CREB3, CREB3L1, CREB3L2, CREB5, CRX, CTCF, CTCFL, CUX1, CUX2, DBP, DBX1, DBX2, DLX1, DLX2, DLX3, DLX4, DLX5, DLX6, DMBX1, DMRT1, DMRT2, DMRT3, DMRTA1, DMRTA2, DMRTC2, DOBOX4, DOBOX5, DPRX, DRGX, DUX4, DUXA, E2F, E2F2, E2F7, E2F8, E4F1, EBF1, EGR1, EGR3, EGR4, EHF, ELF1, ELF2, ELF3, ELF4, ELF5, ELK3, ELK4, EMX1, EMX2, EN1, EN2, EOMES, EP300, ERF, ERG, ESR2, ESRRA, ESRRB, ESRRG, ESX1, ETS, ETV1, ETV2, ETV3, ETV4, ETV5, ETV6, ETV7,	ENCODE, FIMO PWM scan

EVX1, EVX2, FEV, FIGLA, FLI1, FOX,
FOXA, FOXB1, FOXC1, FOXC2, FOXD1,
FOXD2, FOXD3, FOXF1, FOXF2,
FOXG1, FOXI1, FOXJ1, FOXJ2, FOXJ3,
FOXK1, FOXL1, FOXM1, FOXN1,
FOXO1, FOXO3, FOXO4, FOXO6,
FOXP1, FOXP3, FOXQ1, GATA, GBX1,
GBX2, GCM, GCM1, GCM2, GFI1,
GFI1B, GLI, GLI2, GLIS1, GLIS2, GLIS3,
GMEB1, GMEB2, GRHL1, GSC, GSC2,
GSX1, GSX2, GTF2A, GTF2I, GZF1,
HAND1, HBP1, HDAC2, HDX,
HERPUD1, HES1, HES5, HES7, HESX1,
HEY1, HEY2, HF1H3B, HIC1, HIC2,
HIF1A, HINFP, HLF, HLTF, HLX,
HMBOX1, HMGA1, HMGN3, HMX1,
HMX2, HMX3, HNF1, HNF1A, HNF1B,
HNF4, HOMEZ, HOXA1, HOXA2,
HOXA3, HOXA4, HOXA5, HOXA6,
HOXA7, HOXA9, HOXA10, HOXA11,
HOXA13, HOXB2, HOXB3, HOXB4,
HOXB5, HOXB6, HOXB7, HOXB8,
HOXB9, HOXB13, HOXC4, HOXC5,
HOXC6, HOXC8, HOXC9, HOXC10,
HOXC11, HOXC12, HOXC13, HOXD1,
HOXD3, HOXD8, HOXD9, HOXD10,
HOXD11, HOXD12, HOXD13, HSF,
HSF2, HSF4, HSFY2, ID4, IKZF1, IKZF2,
IKZF3, INSM1, IRF, IRF4, IRF6, IRX2,
IRX3, IRX4, IRX5, IRX6, ISL2, ISX,
ITGB2, JDP2, KLF4, KLF7, KLF12,
KLF13, KLF14, KLF16, LBX2, LHX1,
LHX2, LHX3, LHX4, LHX5, LHX6, LHX8,
LHX9, LMO2, LMX1A, LMX1B, MAF,
MAFF, MAFG, MAZ, MEF2, MEF2B,
MEF2D, MEIS1, MEIS2, MEIS3, MEOX1,
MEOX2, MESP1, MGA, MIXL1, MLX,
MLXIPL, MNT, MNX1, MSC, MSX1,
MSX2, MTF1, MXI1, MYB, MYBL1,
MYBL2, MYC, MYCN, MYF, MYF6,
MYOD1, MYOG, MZF1, NANOG,
NEUROD2, NEUROG2, NFAT, NFAT5,
NFATC1, NFATC2, NFE2, NFE2L1,
NFE2L2, NFIA, NFIB, NFIC, NFIL3,
NFIX, NFKB, NFY, NHLH1, NKX1-1,
NKX1-2, NKX2-1, NKX2-2, NKX2-3,

NKX2-4, NKX2-5, NKX2-6, NKX2-8,
NKX3-1, NKX3-2, NKX6-1, NKX6-2,
NKX6-3, NOBOX, NOTO, NR1H, NR1H4,
NR2C2, NR2E1, NR2E3, NR2F2, NR2F6,
NR3C1, NR3C2, NR4A, NR5A1, NR5A2,
NR6A1, NRF1, NRL, OBOX1, OBOX2,
OBOX3, OBOX5, OBOX6, OLIG1,
OLIG2, OLIG3, ONECUT1, ONECUT2,
ONECUT3, OSR1, OSR2, OTP, OTX,
OTX1, OTX2, OVOL2, PATZ1, PAX1,
PAX2, PAX3, PAX4, PAX5, PAX6, PAX7,
PAX8, PAX9, PBX1, PBX3, PDX1,
PHOX2A, PHOX2B, PITX1, PITX2,
PITX3, PKNOX1, PKNOX2, PLAG1,
PLAGL1, POU1F1, POU2F2, POU2F3,
POU3F1, POU3F2, POU3F3, POU3F4,
POU4F1, POU4F2, POU4F3, POU5F1,
POU6F1, POU6F2, PPARA, PRDM1,
PRDM4, PROP1, PROX1, PRRX1,
PRRX2, PTEN, PTF1A, RAD21, RAR,
RARA, RARB, RARG, RAX, RAX2,
RBPJ, REL, REST, RFX2, RFX3, RFX5,
RFX7, RHOXF1, RHOXF2, RORA,
RREB1, RUNX, RUNX1, RUNX2,
RUNX3, RXRA, RXRB, RXRG, SCRT1,
SCRT2, SEF1, SETDB1, SHOX, SHOX2,
SIN3A, SIRT6, SIX5, SMAD, SMAD3,
SMAD4, SMARC, SMC3, SNAI2, SOX,
SOX1, SOX2, SOX3, SOX4, SOX5,
SOX7, SOX8, SOX9, SOX10, SOX11,
SOX12, SOX13, SOX14, SOX15,
SOX17, SOX18, SOX21, SOX30, SP1,
SP2, SP4, SP8, SP100, SPDEF, SPI1,
SPIB, SPIC, SPZ1, SREBP, SRF, SRY,
STAT, T, TAL1, TATA, TBR1, TBX1,
TBX2, TBX4, TBX5, TBX15, TBX19,
TBX20, TBX21, TCF3, TCF4, TCF7,
TCF7L1, TCF7L2, TCF12, TCF21,
TEAD1, TEAD2, TEAD3, TEAD4, TEF,
TFAP2, TFAP2B, TFAP2E, TFAP4,
TFCP2, TFCP2L1, TFE, TFE3, TFEB,
TFEC, TGIF1, TGIF2, TGIF2LX, THAP1,
THRA, THRB, TLX2, TOPORS, TP53,
TP63, TP73, TRIM28, UNCX, VAX1,
VAX2, VDR, VENTX, VSX1, VSX2, WT1,
XBP1, YY1, YY2, ZBED1, ZBTB3,

ZBTB6, ZBTB7A, ZBTB7C, ZBTB12, ZBTB14, ZBTB16, ZBTB18, ZBTB33, ZBTB49, ZEB1, ZFX, ZIC1, ZIC2, ZIC3, ZIC4, ZKSCAN3, ZNF8, ZNF35, ZNF75A, ZNF143, ZNF219, ZNF232, ZNF263, ZNF281, ZNF282, ZNF350, ZNF354C, ZNF384, ZNF410, ZNF423, ZNF524, ZNF589, ZNF628, ZNF652, ZNF691, ZNF713, ZNF740, ZNF784, ZSCAN4, ZSCAN16, ZSCAN26

Table S4: Genome-wide performance of algorithms, trained on 500,000 CpGs, for predicting methylation values.

Algorithm	RMSE (all)	RMSE (int.)	AUROC	AUPRC	Accuracy	Resources	Time (hrs)
BoostMe	0.09 ± 0.005	0.13 ± 0.006	0.99 ± 0.002	0.99 ± 0.0005	0.96 ± 0.005	16 CPUs	0.06 ± 0.02
Random Forests	0.09 ± 0.005	0.13 ± 0.006	0.99 ± 0.002	0.99 ± 0.0005	0.96 ± 0.005	16 CPUs	0.83 ± 0.06
DeepCpG	0.18 ± 0.008	0.29 ± 0.009	0.94 ± 0.004	0.98 ± 0.002	0.91 ± 0.009	1 GPU	66 ± 13

Metrics are averaged across tissues and samples. RMSE, root-mean-squared error; int., intermediate beta values, defined as having a sample average methylation between 0.2 and 0.8; AUROC, area under the receiver operating characteristic curve; AUPRC, area under the precision-recall curve. Time is the average number of computational hours it took to train on all samples within a tissue.

Table S5: RMSE performance of BoostMe and random forests improves when training on continuous values.

Algorithm	RMSE (bin.)	RMSE (cont.)	AUROC (bin.)	AUROC (cont.)	AUPRC (bin.)	AUPRC (cont.)	Accuracy (bin.)	Accuracy (cont.)
BoostMe	0.16	0.09	0.99	0.99	0.99	0.99	0.96	0.96
Random Forests	0.16	0.09	0.99	0.99	0.99	0.99	0.96	0.96
DeepCpG	0.18	0.18	0.94	0.94	0.98	0.98	0.91	0.91

RMSE - root-mean-squared error, AUROC - area under the receiver operating characteristic curve, AUPRC - area under the precision-recall curve, bin. - performance when training on binary methylation values, cont. - performance when training on continuous methylation values.

Table S6: Top 100 transcription factors ranked in descending order as reported by BoostMe, trained only using TFBS features.

Adipose NGT	Adipose T2D	Muscle NGT	Muscle T2D	Islet
EP300	EP300	REST	REST	EP300
REST	REST	ETS	ETS	REST
ETS	ETS	RAD21	RAD21	ETS
PAX5	PAX5	PAX5	PAX5	HEY1
ATF3	ATF3	E2F	ATF3	E2F
E2F	E2F	ATF3	E2F	PAX5
RXRA	RXRA	RXRA	EGR1	HINFP
RAD21	RAD21	RFX5	RFX5	ATF3
HINFP	RFX5	STAT	STAT	RFX5
RFX5	HINFP	EGR1	HNF4	RAD21
HEY1	HEY1	IRF	NR3C1	RXRA
EGR1	EGR1	GATA	RXRA	STAT
STAT	STAT	EP300	GATA	EGR1
NR3C1	NR3C1	HNF4	IRF	BDP1
HSF	IRF	NR3C1	HSF	HSF
IRF	HSF	HSF	EP300	ZBTB33
BDP1	BDP1	BDP1	BDP1	MYBL2
MYBL1	TFAP2	HINFP	MEF2	BHLHE40
GATA	GATA	MEF2	MYBL1	NR3C1
TFAP2	HNF4	MAF	YY1	GATA
HNF4	IRF4	YY1	HINFP	PAX4
IRF4	MYB	MYBL1	MAF	IRF
PAX4	MYBL1	SMAD3	SMAD3	YY1
YY1	PAX4	MYC	CUX1	IRF4
MYB	MEF2	TFAP2	MYC	MYBL1
GFI1	HSFY2	CUX1	CEBPB	MYB
HSFY2	YY1	NFIC	PAX4	GFI1
SPDEF	GFI1	PAX4	GFI1	CUX1
MEF2	BHLHE40	ESRRA	ZSCAN4	ZNF143
CUX1	SPDEF	ZSCAN4	BCL	CACBP
SMAD3	SMAD3	GFI1	FOXJ3	TFAP2
BHLHE40	KLF4	CTCF	HSFY2	CTCF
CTCF	NFATC1	HSFY2	TFAP2	HOMEZ
NFIC	ZBTB33	CEBPB	NFIC	ZBTB14
RUNX3	MYBL2	BCL	XBP1	MEF2
ZBTB33	XBP1	GMEB2	CTCF	KLF4
NFATC1	CUX1	MYBL2	ESRRA	SMAD3
MAF	MAF	FOXJ3	IRF4	NFATC1
GMEB2	GMEB2	MYB	SPDEF	HSFY2
KLF4	CTCF	SPDEF	MYB	FOXJ3
BARHL1	NFY	TBX1	HEY1	HIC2
FOXN1	FOXN1	XBP1	SP1	TBX5

NFY	ZNF524	IRF4	GMEB2	NFIC
HOMEZ	RUNX3	TFCP2	TBX1	GMEB2
XBP1	TATA	RUNX2	TFCP2	SMC3
VDR	NFIC	NFY	HOMEZ	RUNX2
PAX3	PAX3	NFATC1	FOXN1	SPDEF
PTF1A	BCL6B	HOMEZ	HMX3	HNF4
ZNF524	AHR	ZNF713	BCL6B	BARHL2
GRHL1	VDR	PAX3	RUNX2	TCF12
ZNF713	HOMEZ	FOXN1	NFATC1	GLI
MYBL2	CEBPB	FOXK1	NHLH1	FOXN1
FOXC1	PTF1A	TBX20	TATA	NFY
IKZF2	TBX5	SOX9	NFY	TBX4
TATA	GRHL1	TATA	SOX10	FOXC1
NFKB	RUNX2	RHOXF1	TEAD1	TATA
HMGN3	ZNF713	THAP1	MYBL2	BARHL1
ESRRA	FOXJ3	AHR	VDR	PTF1A
CEBPB	ZNF143	ZBTB33	RHOXF1	SP1
HOXA13	IKZF2	SP1	TP53	HDAC2
TBX5	ZBTB14	FOXC1	ZNF282	IKZF2
TFCP2	HMGN3	BARHL1	NFKB	ZNF713
ZBTB14	HOXA13	MNT	ZNF713	CENPB
BCL6B	MYC	OTX1	ZBTB33	HMGN3
AHR	BCL	HEY1	BARHL2	GRHL1
MYC	TFCP2	NFKB	AHR	NFKB
ZNF143	CACBP	BCL6B	TBX20	PAX3
CACBP	BARHL2	TEAD1	ARHGEF12	HIC1
BCL	ESRRA	HOXA5	SIX5	ZSCAN4
HDAC2	FOXC1	ZNF282	BPTF	CHD2
TBX1	NFKB	BARHL2	IKZF2	XBP1
ZNF282	HDAC2	AIRE	PAX3	RUNX3
CENPB	ZNF282	TP53	THAP1	NANOG
ARHGEF12	NFAT	PTF1A	MEIS1	POU2F2
SP1	SOX4	NHLH1	ZBTB6	INSM1
RUNX2	MNT	BPTF	PRDM1	SP8
TBX4	HIC2	VDR	OTX1	NFAT
NFAT	ARHGEF12	TFAP4	GRHL1	MYC
HIC2	TP53	SIX5	PTF1A	ZNF524
ZSCAN4	PRDM1	RARA	FOXA	AHR
FOXK1	NHLH1	RBPJ	RORA	BCL6B
BARHL2	RHOXF1	ZNF524	CHD2	SREBP
HOXC12	ZSCAN4	ARHGEF12	FOXC1	ZNF384
ZBTB7A	CENPB	IKZF2	AIRE	VDR
ZNF384	ZNF384	HDAC2	HOXA5	TCF3
PRDM1	ZBTB6	NFAT	NFAT	TCF4
PROX1	BARHL1	ZNF384	RARG	ARHGEF12
ZBTB6	HIF1A	FOXG1	HIC1	SPI1

SP2	ESRRG	SOX8	ZNF524	PROX1
MNT	GLI	GRHL1	HOXA13	MAF
SP8	HOXC12	HIC1	ZBTB7A	ZNF282
NHLH1	BPTF	EBF1	TFAP4	NRF1
THAP1	ZKSCAN3	HOXA13	FOXG1	ZBTB7A
BPTF	SP2	CCDC6	CRX	CCDC6
ELF1	TBX4	NKX2-8	RUNX3	TFCP2
SIX5	HSF2	ESRRG	PROX1	BCL
HIF1A	PROX1	HMGN3	SOX9	RFX7
FOXJ3	TBX1	SOX10	HOXA11	TP53
SOX4	ELF1	ZBTB12	PAX6	ZBTB3
TP53	ZBTB7A	GZF1	BHLHE40	HNF1

Table S7: Top 100 transcription factors ranked in descending order as reported by random forests trained only using TFBS features.

Adipose NGT	Adipose T2D	Muscle NGT	Muscle T2D	Islet
REST	REST	REST	REST	EP300
EP300	EP300	EP300	EP300	REST
RAD21	RAD21	RAD21	RAD21	BHLHE40
BHLHE40	BHLHE40	E2F	E2F	E2F
E2F	ETS	ETS	ETS	YY1
ETS	E2F	YY1	YY1	RAD21
YY1	YY1	CTCF	CTCF	ETS
CTCF	CTCF	BHLHE40	EGR1	BCL
ZNF143	ZNF143	EGR1	BHLHE40	HEY1
HEY1	HEY1	ZNF143	ZNF143	ZNF143
TFAP2	TFAP2	TFAP2	TFAP2	CTCF
BCL	BCL	BCL	BCL	TFAP2
EGR1	EGR1	ATF3	ATF3	EGR1
ATF3	ATF3	PAX5	PAX5	ATF3
SIN3A	SIN3A	HEY1	HEY1	SIN3A
PAX5	PAX5	SIN3A	SIN3A	ELF1
ELF1	ELF1	MYC	MYC	PAX5
MYC	BDP1	TATA	TATA	MYC
BDP1	MYC	ELF1	NR3C1	HINFP
TATA	TATA	NR3C1	ELF1	BDP1
NR3C1	NR3C1	BDP1	BDP1	TATA
HINFP	HINFP	ESRRA	ESRRA	NR3C1
ESRRA	ESRRA	SP1	SP1	ESRRA
SP1	SP1	RXRA	RXRA	NRF1
RXRA	RXRA	HINFP	HINFP	POU2F2
POU2F2	POU2F2	IRF	IRF	RXRA
NRF1	NRF1	PAX4	AP1	SP1
AP1	AP1	STAT	PAX4	PAX4
PAX4	PAX4	AP1	STAT	CHD2
HIC1	HIC1	NRF1	HNF4	SRF
IRF	IRF	HIC1	NRF1	AP1
NFE2	STAT	HNF4	HIC1	HIC1
STAT	NFE2	POU2F2	POU2F2	NFE2
SETDB1	CHD2	SETDB1	SETDB1	SETDB1
CHD2	SETDB1	NFE2	NFE2	IRF
HNF4	HNF4	CHD2	CHD2	STAT
SPI1	SPI1	SPI1	SPI1	HNF4
SRF	SRF	SRF	SRF	SPI1
EBF1	EBF1	EBF1	EBF1	ZBTB33
ZBTB33	ZBTB33	ZBTB33	ZBTB33	EBF1
ZBTB14	ZBTB14	TCF12	ZBTB14	ZBTB14
TCF12	TCF12	ZBTB14	TCF12	SMC3

SMC3	SMC3	SMC3	SMC3	TCF12
ZIC3	ZIC3	RFX5	RFX5	ZIC3
HDAC2	HDAC2	NFIC	HDAC2	HDAC2
NFKB	NFKB	HDAC2	NFIC	NFKB
NHLH1	NHLH1	GATA	GATA	NHLH1
ZBTB7A	ZBTB7A	NFKB	NHLH1	ZBTB7A
VDR	VDR	NHLH1	NFKB	VDR
NFIC	NFIC	MAF	MAF	NFIC
RFX5	RFX5	ZBTB7A	ZBTB7A	RFX5
SP4	GATA	ZIC3	VDR	GATA
GATA	SP4	VDR	ZIC3	SP4
ZIC1	ZIC1	PBX3	PBX3	MAF
PBX3	PBX3	SP4	SP4	ZIC1
RUNX3	RUNX3	MYB	MYB	PBX3
MAF	MAF	RUNX3	RUNX3	MTF1
EGR3	EGR3	TFCP2	TFCP2	RUNX3
TFCP2	TFCP2	ZIC1	HSF	EGR3
WT1	WT1	AHR	EGR3	TFCP2
MYB	MYB	HSF	ZIC1	MYB
MTF1	MTF1	EGR3	RUNX2	WT1
RUNX2	RUNX2	RUNX2	AHR	TCF3
AHR	AHR	SP2	TCF3	ZFX
HF1H3B	TCF3	TCF3	MTF1	HF1H3B
TCF3	HSF	MTF1	SPDEF	TP53
HSF	HF1H3B	SPDEF	TP53	TFAP4
EGR4	EGR4	TFAP4	SP2	AHR
SP2	SP2	WT1	WT1	RUNX2
ZFX	TP53	TP53	TFAP4	SP2
SPDEF	ZFX	PAX2	PAX2	TFAP2B
TFAP2B	TFAP2B	EGR4	EGR4	SPDEF
TP53	SPDEF	MEF2	MEF2	SPZ1
TFAP4	TFAP4	CUX1	HF1H3B	EGR4
ZIC4	SPZ1	SMAD3	SMAD3	NR1H
SPZ1	ZIC4	HF1H3B	CEBPB	PLAG1
NR1H	ZNF219	SREBP	CUX1	ZIC4
ZNF219	NR1H	TFAP2B	NR1H	ZNF219
PAX2	PAX2	NR1H	MYBL1	HSF
RREB1	RREB1	ZFX	RARA	RREB1
ZNF281	ZNF281	RARG	ZFX	PAX2
GLI2	PLAG1	RARA	TFAP2B	PPARA
PLAG1	GLI2	RREB1	SREBP	ZBTB3
SREBP	PPARA	MYBL1	RARG	GLI2
PPARA	SREBP	SPZ1	THAP1	NANOG
THAP1	NANOG	CEBPB	ZNF281	SREBP
PLAGL1	TEAD2	THAP1	SPZ1	PLAGL1
TEAD2	THAP1	ZIC4	ZIC4	CACD

KLF4	PLAGL1	PAX6	RREB1	ZNF281
SMAD3	RARA	XBP1	XBP1	MYOD1
ZBTB3	KLF4	GLI2	PPARA	KLF12
CEBPB	ZNF524	ZNF281	PAX6	THAP1
NANOG	ZBTB3	PAX3	GMEB2	TEAD2
RARA	CEBPB	MYBL2	GLI2	RARA
ZNF524	CACD	PPARA	ZNF219	NR2C2
CACD	PATZ1	GFI1	RFX3	SMAD3
PATZ1	NR2C2	TBX20	TBX20	GCM1
RARG	SMAD3	MYOD1	MYOD1	PATZ1
KLF12	KLF12	KLF4	NANOG	PAX6
KLF7	KLF7	GMEB2	GFI1	ELF2
